# 27‐Hydroxycholesterol represses G9a expression via oestrogen receptor alpha in breast cancer

**DOI:** 10.1111/jcmm.17882

**Published:** 2023-08-23

**Authors:** Ravindran Vini, Asha Lekshmi, Swathy Ravindran, Jissa Vinoda Thulaseedharan, Kunjuraman Sujathan, Arumugam Rajavelu, Sreeharshan Sreeja

**Affiliations:** ^1^ Cancer Research Program, Rajiv Gandhi Centre for Biotechnology (RGCB) Thiruvananthapuram India; ^2^ Research Centre University of Kerala Thiruvananthapuram India; ^3^ Laboratory of Cytogenetics and Molecular Diagnostics Division of Cancer Research, Regional Cancer Centre Thiruvananthapuram India; ^4^ Achutha Menon Centre for Health Science Studies (AMCHSS) Sree Chitra Tirunal Institute for Medical Sciences and Technology Thiruvananthapuram India; ^5^ Health Software Technology Group, Centre for Development of Advanced Computing (CDAC) Thiruvananthapuram India; ^6^ Department of Biotechnology, Bhupat & Jyoti Mehta School of Biosciences Indian Institute of Technology Madras Chennai India

**Keywords:** breast cancer, epigenetics, histone methylation, metabolites, oestrogen receptor alpha

## Abstract

27‐hydroxycholesterol (27‐HC) is a cholesterol metabolite and the first discovered endogenous selective estrogen receptor modulator (SERM) that has been shown to have proliferative and metastatic activity in breast cancer. However, whether 27‐HC metabolite modulates the epigenetic signatures in breast cancer and its progression remains unclear. The current study, reports that 27‐HC represses the expression of euchromatic histone lysine methyltransferase G9a, further reducing di‐methylation at H3K9 in a subset of genes. We also observed reduced occupancy of ERα at the G9a promoter, indicating that 27‐HC negatively regulates the ERα occupancy on the *G9a* promoter and functions as a transcriptional repressor. Further, ChIP‐sequencing for the H3K9me2 mark has demonstrated that 27‐HC treatment reduces the H3K9me2 mark on subset of genes linked to cancer progression, proliferation, and metastasis. We observed upregulation of these genes following 27‐HC treatment which further confirms the loss of methylation at these genes. Immunohistochemical analysis with breast cancer patient tissues indicated a positive correlation between G9a expression and CYP7B1, a key enzyme of 27‐HC catabolism. Overall, this study reports that 27‐HC represses G9a expression via ERα and reduces the levels of H3K9me2 on a subset of genes, including the genes that aid in breast tumorigenesis and invasion further, increasing its expression in the breast cancer cells.

## 1 INTRODUCTION

27‐Hydroxycholesterol (27‐HC) is an oxysterol and the most abundant cholesterol metabolite in the bloodstream. It is the first identified endogenous selective oestrogen receptor modulator (SERM), implying that its actions are highly tissue‐specific.^1^ 27‐HC promotes atherosclerosis,^2^ reduces bone density,^3^ contributes to brain‐related pathogenesis,^4^ aids in thyroid cancer^5^ and breast cancer proliferation.^6^ 27‐HC promotes breast cancer proliferation via ERα and induces metastasis via LXRα.^6^ The metabolite further modulates γδ‐T cells,^7^ impairs T cell expansion,^6^ and promotes the secretion of extracellular vesicles, including exosomes^7^ from polymorphonuclear neutrophils, which in turn promotes tumour growth and metastasis. At the clinical level, the role of 27‐HC is yet to be elucidated. CYP7B1, the key enzyme responsible for the catabolism of 27‐HC, is associated with increased recurrence‐free survival,^8^ whereas the expression of the 27‐HC synthesising enzyme CYP27A1 is linked to unfavourable tumour characteristics.^9^, ^10^, ^11^, ^12^ A study has demonstrated that the serum 27‐HC level does not correlate with its levels in breast tumours, unlike in normal breast tissue, suggesting a possible association of dysregulation of 27‐HC metabolism in breast cancer cells.^12^ A recent study has shown that elevated levels of circulating 27‐HC are associated with a reduced risk of breast cancer in postmenopausal women.^10^ However, it is important to note that it is not the level of circulating 27‐HC but the level of intratumoral 27‐HC that provides a better picture of its tumorigenicity.

Aberrant epigenomic signatures play a pivotal in breast cancer development, progression and therapeutic resistance.^13^, ^14^, ^15^ During the early stages of carcinogenesis, alterations in chromatin structure via DNA hypermethylation/hypomethylation and post‐translational modifications of histones affect cellular phenotypes and favour the oncogenic reprogramming of tumour progenitor cells. These changes promote the acquisition of uncontrolled self‐renewal properties.^15^ In the later stages of cancer progression, additional epigenetic changes, along with subclonal mutations and signals from the microenvironment, dictate the cancer cell phenotype, affect the metastatic propensity of the tumour, and confer endocrine therapy resistance.^13^, ^15^ Other epigenetic modifiers, such as noncoding RNAs, especially micro RNAs and long noncoding RNAs, and changes in histone marks, are also known to contribute to breast cancer progression.^14^ Attempts are being made to understand the intricate network of functional interactions among oestrogen receptors, oncogenic transcription and epigenetic machinery, with emphasis on how these factors are assembled upon acute oestrogen exposure, which is one of the known risk factors for breast cancer.^13^ Although it is known to some extent that oestrogen drives breast cancer proliferation, the molecular mechanisms, hierarchical events upon oestrogen and/or ERα ligand exposure, and effects on gene regulation and chromatin organisation at the genomic and epigenomic levels are not well understood.^16^

27‐HC is known to induce cellular transition in breast cancer cells. Nevertheless, whether aberrant epigenome contributes to 27‐HC–mediated cellular and morphological changes and breast cancer progression is unknown. We had earlier reported that 27‐HC induces DNA methylation changes in subsets of genes in breast cancer cells.^17^ In this study, 27‐HC–mediated changes in histone methyltransferases in breast cancer cells were investigated. 27‐HC was found to represses *G9A /EHMT2* expression via ERα in MCF‐7 cells. 27‐HC decreased the occupancy of ERα on the G9a promoter, leading to the downregulation of G9a expression. ERα functioned as a transcriptional activator of G9a and 27‐HC, reversing this process in MCF‐7 cells.

Furthermore, the reduced expression of G9a upregulated the proliferative genes in MCF‐7 cells. In addition, elevated G9a expression was correlated with the increased expression of CYP7B1, the 27‐HC catabolising enzyme, in the tissues of patients with breast cancer in the premenopausal age group. Our data suggest that the local concentration of the metabolite in the microenvironment of breast cancer tissues induces epigenetic alterations and cell proliferation.

## 2 EXPERIMENTAL PROCEDURES

### 2.1 Cell Culture

ER‐positive cell lines, MCF‐7 and T47D cells were used in this study. The cells were cultured in Dulbecco's Modified Eagle Medium (DMEM) with 10% fetal bovine serum (FBS) (Invitrogen) and 1% penicillin and streptomycin (HiMedia) at 37°C with 5% CO2. The cells were transfected at around 70%–80% confluency with lipofectamine 2000 (Invitrogen) according to manufacturer's instruction using ERαsiRNA sc‐29305 with OptiMeM medium followed by medium change after 6 h of transfection. The cells were either treated with 27‐HC (1 μM) or DMSO (0.1%) for various time points. The cells were maintained in phenol‐red free DMEM (Sigma Cat#D1152) supplemented with 5% charcoal‐stripped serum 72 h prior to treatment. The treatments were done in phenol‐red free DMEM supplemented with 5% charcoal‐stripped serum.

### 2.2 RNA isolation, RT‐PCR and qPCR

The MCF‐7 cells were seeded at a cell density of 1 × 10^5^ cells in 60 mm dishes. Total RNA was isolated using TriZol reagent (Sigma). The quality and concentration of RNA was measured with Nanodrop (2000, ThermoFischer). First strand cDNA synthesis was carried out with One Step RT‐PCR Kit Ver.2 (Cat. #RR055A/(TAKARA) using 1 μg of total RNA. The qPCR was carried out as per the manufactures instructions using SYBR Premix Ex Taq II (TAKARA) in a 7500 Real‐Time PCR System (Applied Biosystems). Gene expression levels were calculated by the 2^−ΔΔCt^ method using *GAPDH* as control (Table S1). All the samples were assessed in triplicates and the experiment was done in triplicates.

### 2.3 Western Blot

The cells were seeded at a cell density of 1×10^6^ cells in 100 mm dishes. As the cells attained their morphology, they were treated with 27‐HC at 1 μM for different time points, the cells were seeded in parallel. The cells were scraped and lysed using RIPA buffer NaCl (500 mM) 0.175 g, 1% NP 40,0.5% Sodium deoxycholate, 0.1% SDS, Tris buffer (pH 8.0). Total protein concentration was quantified by Bradford's assay. 40–60 μg of protein was loaded onto 7%–10% SDS—PAGE gel and was subjected to electrophoresis. Proteins were further transferred to PVDF membrane by western blot. The membranes were blocked with 5% BSA for an hour and washed with 1X TBST buffer. The blot was incubated with primary antibody overnight at 4°C. This was followed by wash with 1X TBST. The blots were further incubated with suitable secondary antibody for 90 min. Further, the blots were developed with ECL reagent (Pierce). In the experiment involving proteasomal inhibitor, MG132, the cells were treated with the inhibitor 2 h prior to 27‐HC treatment. The primary antibodies used include, G9a/EHMT2 (Abcam ab185050), p21 (BD Pharmingen 556430), β‐actin (Santa Cruz, SC‐69879), ERα (Santa Cruz, SC‐8002), GAPDH (Santa Cruz, SC‐47724).

### 2.4 Histone extraction

The cells were harvested and washed twice with cold 1X phosphate‐buffered saline (PBS). Cell lysis buffer (10 mM Tris pH 8.0, 1.5 mM MgCl_2_, 1 mM DTT, 1X Protease Inhibitor cocktail, 1 M NaCl_2_) was added to the cell pellets and incubated for 30 min at 4°C in tube rotator and centrifuged at 10,000 ***g*** for 10 min at 4°C. Histones were then extracted from the nuclei pellet with 0.4 N H_2_SO_4_ for 2 h and precipitated by 33% TCA at 4°C overnight. The histone proteins were washed using ice‐cold acetone. After centrifugation at 16,000 ***g*** for 5 min at 4°C, acetone was discarded.

The histone proteins were dried at room temperature for 20 min and reconstituted in distilled water. The concentration of extracted histone was measured by nanodrop. The purity and quality of histones were affirmed by 16% SDS‐PAGE analysis. To assess the H3K9me2 mark status on the histones, the histone proteins were separated on 16% SDS‐PAGE, followed by western blot and blocked with 5% BSA. Further, the membrane was probed with anti‐H3K9me2 polyclonal antibody (Diagenode Cat no: C15410060), or anti‐H3 antibody (C15210011, Diagenode) overnight at 4°C and incubated with appropriate secondary antibodies and developed using ECL reagent (Bio‐Rad, 1705060).

### 2.5 Chromatin immunoprecipitation assay

The cells were seeded at a cell density of 1 × 10^6^ cells in 100 mm dishes. As the cells attained their morphology, they were treated with 27‐HC at 1 μM for 72 h. After the treatment period, the cells were cross‐linked for 10 min at room temperature with 1% formaldehyde and then quenched with 0.125 M of glycine for 5 min. The cells were resuspended with 1 mL lysis buffer (10 mM Tris–HCl pH 8.0, 20% NP40, 3 mM MgCl_2_) and incubated on ice for 10 min. Next, sucrose solution (0.25 M sucrose, 50 mM Tris pH 8.0, 10 mM EDTA) was added and centrifuged at 12,000 rpm, 4°C for 5 min. The supernatant was discarded and pellet was resuspended in 1X PBS. The chromatin shearing was carried out by sonication (Bioruptor UCD 200, Diagenode). For 22 cycles, each cycle with a pulse of 30 s of sonication followed by a 30‐s rest period. For qPCR analysis, 25 μg sheared chromatin was incubated with 1.8 μg ERα antibody, and 400‐μL ChIP buffer (20 mM Tris–HCl pH 8.0, 100 mM NaCl, 6 mM EDTA, 1% Triton X‐100) overnight at 4°C for both control and 27‐HC treated chromatin. The following day, 15 μL of protein A dynabeads was added to the complexes and incubated for 3–4 h. Then the bound complexes were washed with wash buffer I thrice (20 mM Tris–HCl pH 8.0, 180 mM NaCl, 6 mM EDTA, 1% Triton X‐100) followed by wash with ChIP buffer. The immune complexes were eluted with 100 μL elution buffer (1% SDS, 50 mM NaHCO3) and DNA protein complexes were reverse crosslinked at 65°C for 45 min and then purified with DNA purification kit (Macherey‐Nagel, 740609.50). The input was processed by mixing chromatin from both treatments to achieve equal concentration. The qPCR was carried out using *G9a* specific promoter primers. The resulting signals were normalized to input DNA.

For ChIP sequencing, 1.6 μg of sheared chromatin was incubated with 1.8 μg H3K9me2 antibody, and 250 μL ChIP buffer (20 mM Tris–HCl pH 8.0, 100 mM NaCl, 6 mM EDTA, 1% Triton X‐100) overnight at 4°C for both DMSO and 27‐HC treated samples. Next day, 15 μL of protein A dynabeads were added to the immuno‐complexes and incubated for 3–4 h. The samples were washed with ChIP buffer followed by wash buffer I (20 mM Tris–HCl pH 8.0, 180 mM NaCl, 6 mM EDTA, 1% Triton X‐100). The immune complexes were eluted with 100 μL elution buffer (1% SDS, 50 mM NaHCO3) at 65°C for 45 min. and then DNA was purified with DNA purification kit (GE healthcare).

### 2.6 ChIP sequencing

ChIP DNA and the input DNA were subjected to end repair and tailing with dA‐tail followed by ligation of adapter sequences. These adapter ligated fragments are then size selected using SPRI bead, followed by size selected fragments are indexed during limited cycle PCR to generate final libraries for paired‐end sequencing. The resulting libraries are quantified and subjected to sequenced on Illumina HiSeq 2500/4000 system to generate 2 × 50 bp sequence reads.

### 2.7 Data processing

The fastq files were subjected to quality and the following parameters from fastq file were checked to confirm the quality. The base quality score distribution, sequence quality score distribution, average base content per read, PCR amplification issues and analysis of over‐represented sequences. The adapter trimming was performed using Trimmomatic (Ver‐0.36).^18^ The paired‐end reads are aligned to the reference hg19/GRCh37 human genome built. Only uniquely mapped reads were taken further for analysis. To remove any false positive location of binding, the black listed region of the human genome was filtered out and proceed to peak calling using MACS2 (MACS 2.1.3 version).^19^ These peaks are annotated using HOMER (annotatepeaks) and GREAT tool.^20^ Peak annotation visualisation using ChipSeeker R package.^21^ The motif discovery was performed using DREME^22^ and MEME software.^23^ The MEME discovers novel, ungapped motifs in the sequences. DREME discovers short, ungapped motifs that are relatively enriched in the sequences compared with shuffled sequences.

### 2.8 Tissue microarray construction

Sixty‐eight ER‐positive and 19 ER‐negative samples were selected to construct the tissue microarrays (TMAs). The microarray was constructed using Unitma TMA recipient block mould kit Quick Ray™ mould (Unitma Co.). Microarray of 15 tissue cores of 5.0 mm each was prepared for the study. The array was constructed as per the manufacturer's protocol. Recipient paraffin blocks for embedding 15 tissue cores were constructed using Quick Ray mould as instructed. Appropriate tissue areas for preparing the array were selected from an haematoxylin and eosin‐stained slide of the parent block. The tumour area for the study was marked in the haematoxylin and eosin‐stained slide and that area was punched into a tube‐shaped section of 5.0 mm using the quick ray needle. The core was transferred to a predetermined position in the recipient paraffin block.

Histopathological features by haematoxylin and eosin staining of all tumour samples in the microarray was verified. Expression of different proteins of interest ERα, PR, EHMT2/G9A, CYP7B1 and CYP27A1 were analysed by immunohistochemical assay and MACH4 Universal HRP Polymer Detection Kit (BioCare Medical, #BRI4012H) was used for horseradish peroxidase (HRP) DAB mediated detection of staining. For immunostaining, one tumour‐rich section of 4 μm thickness was picked out. The sections were deparaffinized in xylene, rehydrated in graded series of alcohol and antigen retrieved by microwave method as per antibody specification. Endogenous peroxidase activity and non‐specific protein binding was blocked using MACH4 detection kit reagents as per manufacturer's protocol. The sections were incubated with primary antibodies of specified dilutions directed against the following proteins: ER (Santa Cruz Biotechnology; #SC‐8002), PR (Abcam; #32085), EHMT2/G9A (Abcam; #185050), CYP7B1 (Invitrogen; #PA5‐28121) and CYP27A1 (Invitrogen; #PA5‐75271). After overnight incubation with primary antibody at 4°C in humid chamber, the sections were treated with HRP conjugated MACH4 secondary probe for 30 min at room temperature. The presence of protein of interest was visualized using DAB based detection system. The slides were counterstained with haematoxylin and mounted for visualisation and microscopic scoring of staining. The images of immunohistochemical staining of different proteins in tumour tissues were collected using light microscope (Leica DM750, Leica microsystems GmbH) and scored them by perceiving >5 high power fields with roughly 1000 cells. The ERα and PR IHC slides were scored based on Allred scoring system with samples having more than 1% nuclear expression considered as positive. The samples negative for ERα and PR by IHC were grouped as hormone receptor negative breast cancers. For other proteins studied such as EHMT2/G9A, CYP7B1 and CYP27A1 scoring was performed in semi‐quantitative way by H‐score method.

### 2.9 Statistical analysis

Results were expressed as a mean ± standard deviation of three biological replicates. The *p* value was determined using paired two‐sided Student's *t*‐test, one‐way anova or two‐way anova, depending on the experiments. In all the tests, values were statistically significant when *p* ≤ 0.05; *p ≤ 0.05; **p ≤ 0.01 versus control. The Pearson correlation coefficient was used to assess all the correlation in the tumour samples. All statistical analyses were performed using Stata/SE 17.0 and MS excel. Each variable was analysed by using Median test and the data are represented as median and interquartile range.

## 3 RESULTS

### 3.1 27‐HC mediates cellular changes along with the regulation of epigenetic enzymes in ER‐positive breast cancer cells

**FIGURE 1:**
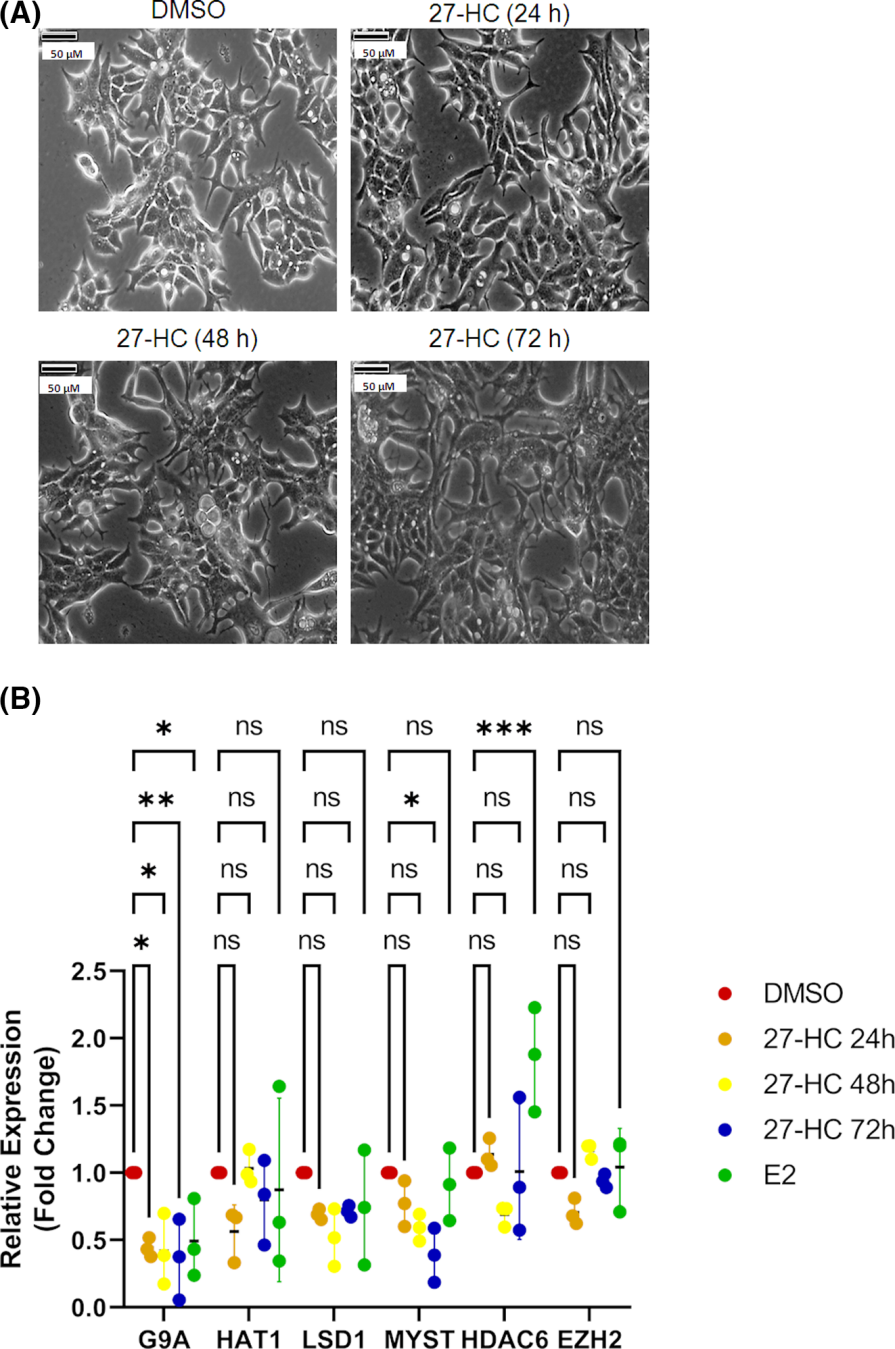
Cellular and transcriptional changes with the regulation of epigenetic enzymes in ER‐positive breast cancer cells: (A) Morphological changes observed in MCF‐7 cells treated with 27‐HC (1 μM) for 24–72 h. (B) The graph represents the qRT‐PCR analysis for the expressing G9A, HAT1, LSD1, MYST, HDAC6 and EZH2 in MCF‐7 cells treated with 27‐HC (1 μM) for 24–72 h. G9a showed consistent downregulation at all time points. The GAPDH was used as normalisation control, and the error bar represents the standard deviation of three independent replicates. The *p* values were calculated using paired *t*‐test, and the differences were considered significant at *p* values *p* < 0.05(*) <0.01(**) <0.001(***).

27‐HC induced epigenetic alterations in breast cancer cells was assessed, given its association with cell proliferation. ER‐positive breast cancer cells were treated with the 27‐HC ligand for 24, 48 and 72 h, and the results showed that 27‐HC induced significant morphological changes in breast cancer cells (Figure 1A). Cellular phenotypic alterations were more evident in case of prolonged exposure to 27‐HC. Subsequently, whether these morphological changes were related to dysregulated epigenetic enzyme expression upon 27‐HC exposure in MCF‐7 cells was assessed. qRT‐PCR analysis of various epigenetic enzymes was performed after the treatment of MCF‐7 cells for 24 h with 27‐HC to determine the potential link between cellular morphological changes and epigenetic alteration. We have analysed the expressions of various epigenetic enzymes such as histone acetyltransferase, histone lysine methyltransferases and DNA methyltransferases. List of histone acetyltransferases: *HAT1* (H4K5/12), *MYST2* (H4K12). List of histone deacetylases: (*HDAC) HDAC1*, *HDAC6*, *HDAC7*. List of histone lysine methyltransferases: *(HKMT) SET9* (K4), *SMYD3* (K4), *SUV39H* (K9), *G9A* (K9), *EZH2* (K27), *DOT1L* (K79), *MLL2* (K4), *MLL2* (K4), *SETD2* (K36), *SETDB1*(K9) histone lysine demethylase (HKDM), *LSD1* (K4), *JMJD3* (K27). List of DNA methyltransferases: (DNMT) *DNMT3A*, *DNMT3B*, and *DNMT1*. The genes that showed differential expression were further analysed for their expression at different time points of 27‐HC treatment, that is, 24, 48 and 72 h. Interestingly, 27‐HC treatment altered the expressions of *G9a*, *HAT1*, *LSD1*, *MYST*, *HDAC6* and *EZH2*, of which *G9a* showed a significant reduction in the presence of 27‐HC at all time points of the treatment (Figure 1B).

### 3.2 27‐HC downregulates G9a expression in MCF‐7 cells

**FIGURE 2:**
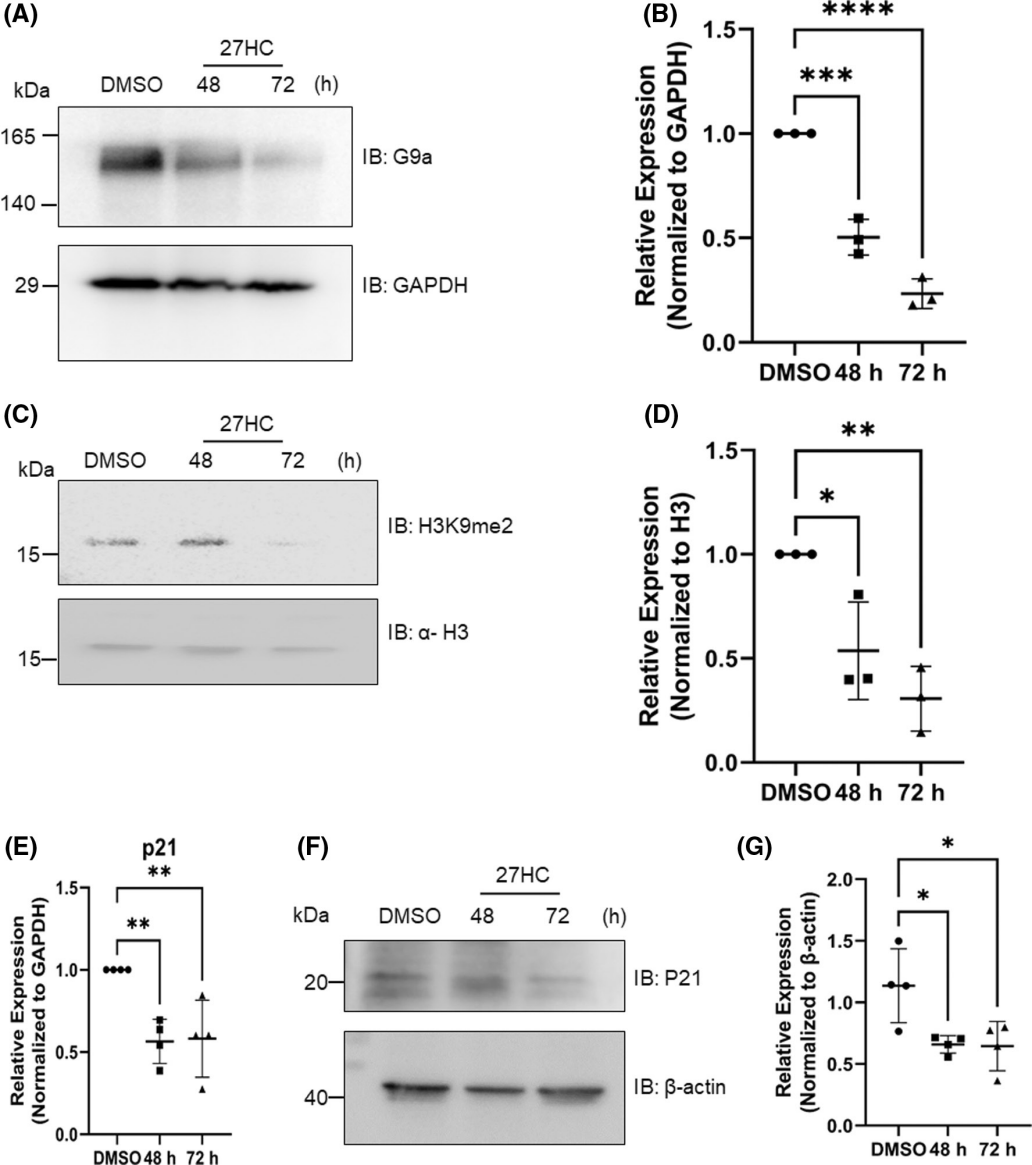
Downregulation of G9a expression and its targets upon 27‐HC treatment: (A) Protein expression analysis by western blot showed a reduction of G9a upon 27‐HC treatment (1 μM). (B) The graph represents the intensity of G9a normalized with the intensity of corresponding GAPDH. (C) H3K9 dimethylation was analysed by western blot, which showed a reduction in agreement with the observation of G9a decrease. (D) The graph represents the intensity of H3K9 dimethylation normalized with the intensity of corresponding Histone H3 levels. (E) p21gene (CDKN1A), a non‐histone target of G9a was checked for its expression by qRT‐PCR. p21 levels were seen to decrease. (F, G). The graph represents the intensity of p21 normalized with intensity of corresponding β‐Actin levels. The western blot analysis of p21 was consistent with its transcriptional expression pattern. The western blot and qRT‐PCR were performed in biological triplicate. The intensity of bands in western blot analysis were measured using ImageJ. The *p* values were calculated using two‐way anova and the differences were considered significant at *p* values *p* < 0.05(*) <0.01(**) <0.001(***).

Of the selected HKMTs, significant downregulation of *G9a* was observed in MCF‐7 cells upon treatment with 27‐HC. G9a is a well‐known euchromatic histone lysine methyltransferase that introduces the H3K9me2 mark on the chromatin and is linked to gene repressor activity.^24^ The expression of the G9a protein after treatment with 27‐HC was assessed, which revealed that G9a levels decreased concurrently at transcript levels at all time points. The highest reduction was observed at 72 h (Figure 2A,B). As G9a is one of the key HKMTs that introduces the H3K9me2 mark on the chromatin, the levels of the H3K9me2 mark on the chromatin isolated from MCF‐7 cells treated with 27‐HC were analysed. In concordance with the downregulation of G9a, a reduction in the H3K9me2 mark was observed after 27‐HC treatment (Figure 2C,D). G9a is known to regulate the expressions of numerous genes. The expression of one of the well‐known G9a targets, *CDKN1A*/p21 gene, was analysed. Studies have shown that G9a can either activate^25^ or repress p21 expression.^26^, ^27^ In this study, 27‐HC was found to downregulate G9a levels, which led to reduced expression of p21 at the transcriptional and translational levels (Figure 2E–G), suggesting that G9a functions as a transcriptional activator of *CDKN1A*/p21 gene expression in MCF‐7 cells. p21 is a two‐faced regulator depending on cell type, cellular localisation, p53 status and the type and level of genotoxic stress.^28^, ^29^ p21 can acquire oncosuppressive properties when it is in a p53‐proficient environment and oncopromoting properties when it is in a p53‐deficient environment.^29^ As MCF‐7 cells are known to contain wild‐type p53, the observed reduction in p21 levels upon 27‐HC treatment may also contribute to the tumorigenic function in cells.

### 3.3 Mechanisms of 27‐HC–mediated suppression of G9a expression

**FIGURE 3:**
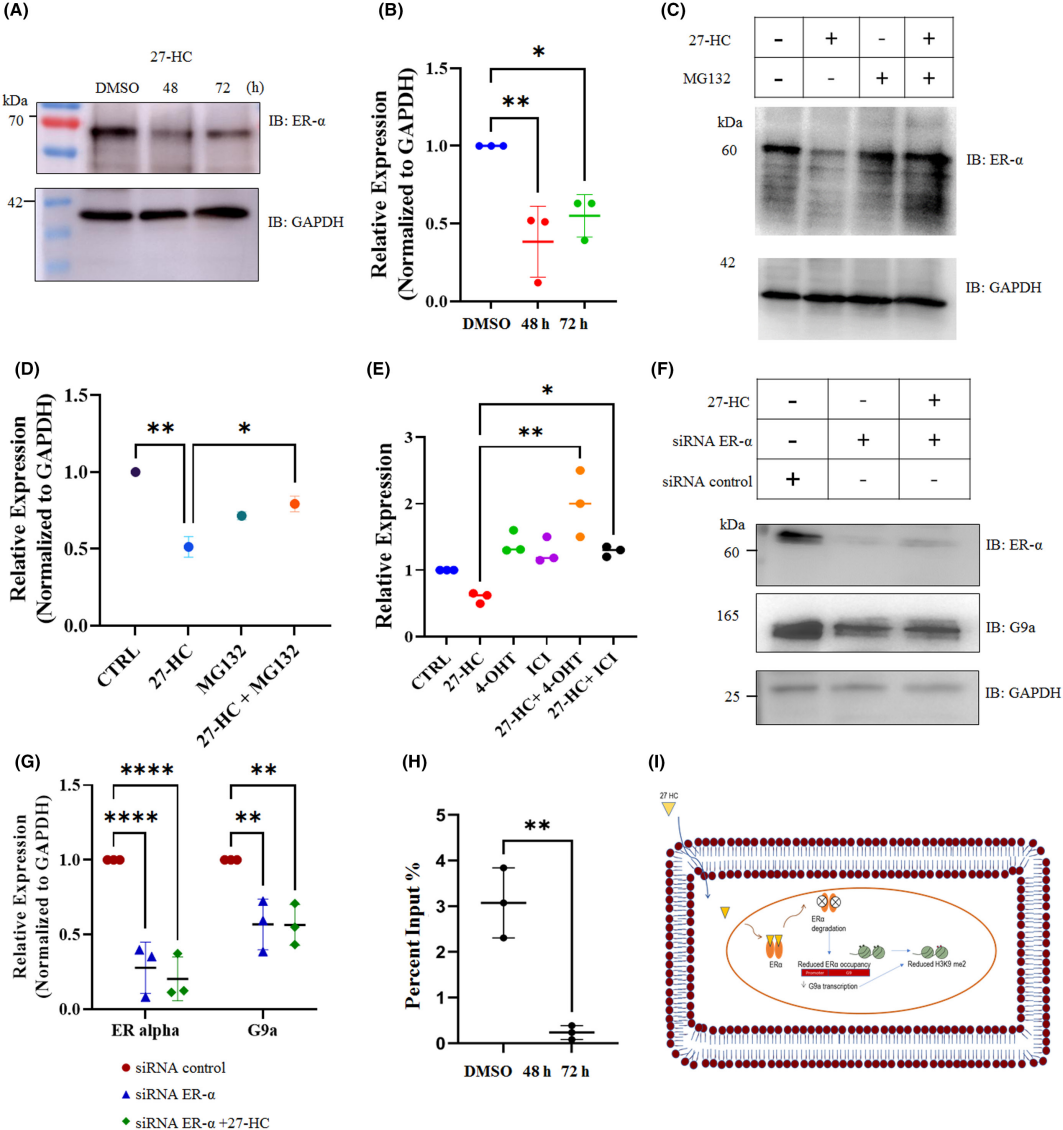
27‐HC mediated suppression of G9a expression.(A) Expression of ERα were analysed by western blot upon 27‐HC treatment. (B) The graph represents the intensity of ERα normalized with intensity of corresponding GAPDH. (C) Expression of ERα were analysed by western blot with or pre‐treatment with MG132 for 2 h followed by 27‐HC treatment. (D) The graph represents the intensity of ERα normalized with intensity of corresponding GAPDH.(E) The graph represents the qRT‐PCR analysis for G9a upon treatment with 4‐hydroxytamoxifen (4‐OHT), ICI‐182780 (ICI), (fulvestrant) in combination with 27‐HC or alone. (F) ERα knockdown reduced G9a expression. MCF‐7 cells were transfected with ERα siRNA or scrambled siRNA and was treated with 27‐HC or DMSO for 48 h. Expression of G9a and ERα were analysed by western blot. (G) The graph represents the intensity of G9a and ERα normalized with intensity of corresponding GAPDH. (H) ChIP‐qPCR was performed upon pull down with ERα to observe the occupancy of ERα at G9a promoter after treating MCF‐7 cells with 27‐HC (1 μM) for 72 h. The G9a promoter specific primers were used for qPCR analysis. There was decrease in the level of G9a promoter fragments after 27‐HC treatment indicating a reduced occupancy of ERα at the G9a promoter. The western blot and qRT‐PCR were performed in biological triplicate. The intensity of bands in western blot analysis were measured using ImageJ. The *p* values were calculated using two‐way anova and the differences were considered significant at *p* values <0.05(*), <0.01(**), <0.001(***). (I) Represents the schematic diagram of the proposed mechanism: 27‐HC bound ERα get degraded which reduces its occupancy at G9a promoter leading to its downregulation and its further consequences.

The mechanisms of 27‐HC–mediated suppression of G9a were studied next. Whether G9a reduction was mediated by the ERα receptor was tested. 27‐HC was observed to reduce ERα, which is in agreement with a previous report (Figure 3A,B).^30^ Furthermore, to ascertain whether ERα degradation is proteasome mediated the cells were treated with proteasomal inhibitor MG132, 2 h before 27‐HC treatment. MG132 treatment was found to reduce ERα degradation and attenuate 27‐HC–mediated ERα degradation (Figure 3C,D). To confirm this finding, the ERα antagonist ICI‐182780 (ICI)/fulvestrant and the SERM, 4‐hydroxytamoxifen were combined with 27‐HC, which resulted in an upregulation of *G9a* gene expression (Figure 3E). Furthermore, ERα was silenced using small interfering RNA in MCF‐7 cells, which led to a significant reduction of G9a in the ERα silenced cells (Figure 3F,G). This finding emphasizes the contribution of ERα in 27‐HC–mediated G9a suppression. In addition, to understand whether ERα directly interacts with G9a promoter in the presence of 27‐HC, a ChIP assay was performed for ERα using cells treated with DMSO or 27‐HC for 72 h. qPCR analysis of the G9a promoter confirmed the enrichment of ERα on the *G9a* promoter in DMSO‐treated cells, whereas 27‐HC treatment reduced the occupancy of ERα on the promoter (Figure 3H,I). These results suggest that the loss of the ERα receptor upon 27‐HC treatment leads to reduced occupancy of ERα on the G9a promoter (Figure 3D). Similar to the reduction of G9a upon ERα silencing (Figure 3F,G), 27‐HC treatment also reduced the expression of G9a. This observation alludes that ERα is a transcriptional activator of G9a. This result was further validated by analysing G9a expression and its downstream target p21 in other ER‐positive cell line, T47D. A similar effect was noted in the expression pattern of G9a and p21 proteins in the presence of 27‐HC in T47D cells (Figure S2). To substantiate our finding, the regulation of G9a in ER‐negative cells (MDA‐MB 231 cells) was tested. The result revealed that the treatment of ER‐negative cells with 27‐HC did not alter the G9a expression (Figure S3), which further confirms the role of ERα‐mediated regulation of G9a by 27‐HC. From these observations, it is evident that a concurrent reduction of ERα and G9a occurs upon 27‐HC treatment; however, the contribution of other factors in regulating G9a expression cannot be ruled out.

### 3.4 H3K9me2 ChIP sequencing identifies the aberrant G9a target genes in 27‐HC–treated cells

**FIGURE 4:**
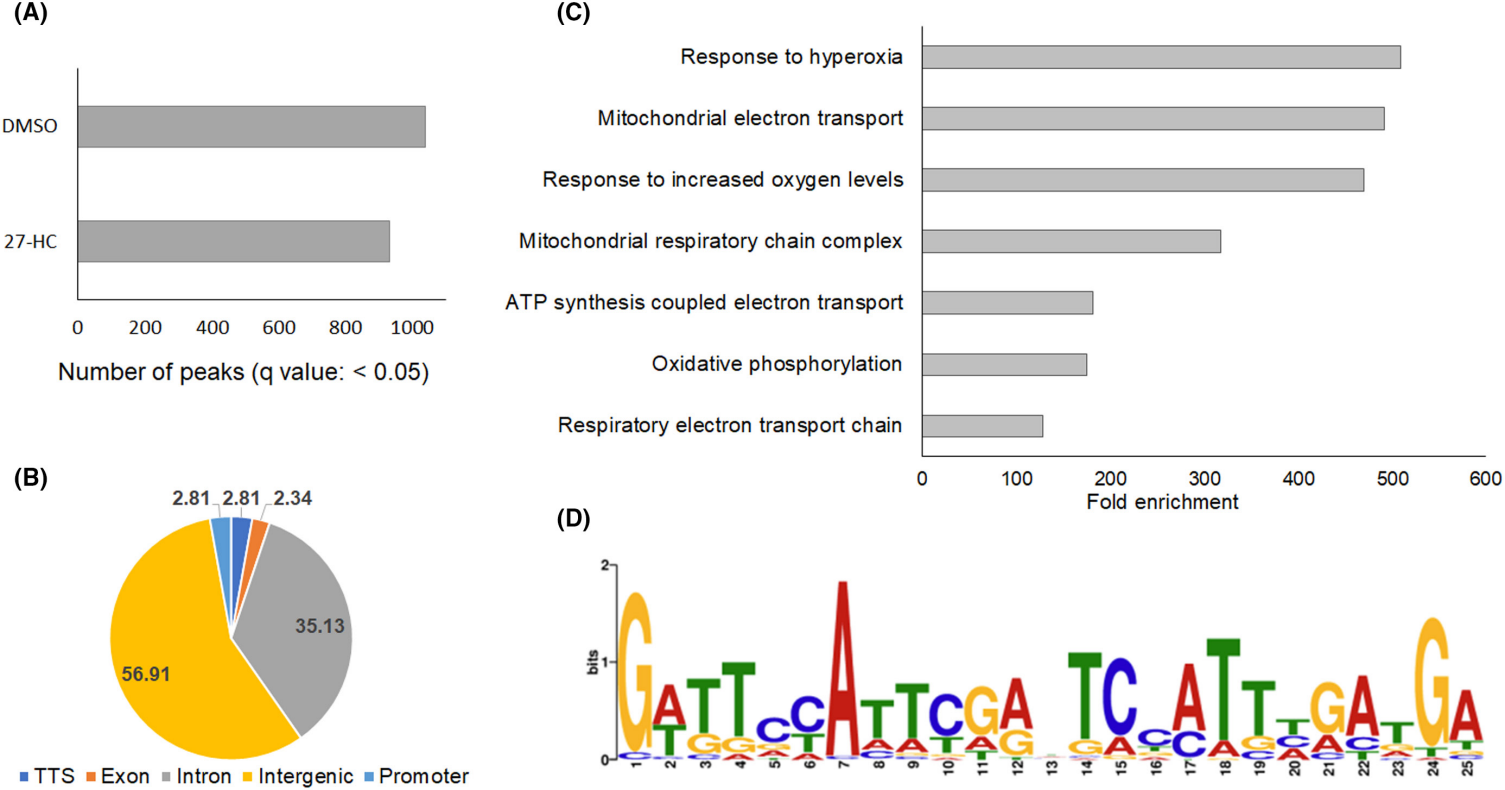
ChIP sequencing analysis of H3K9me2 mark. (A) The bar graph representation of number of ChIP‐sequencing peaks observed in DMSO and 27‐HC treated cells for 72 h. (B) The pie diagram represents the distribution of unique peaks from DMSO treated cells, which are absent in the 27‐HC treated cells. The large number of unique peaks mapped to the intergenic and intron regions of the genes. (C) The GO analysis indicates that the unique ChIP sequencing peaks are clustered to the biological pathways linked to oxygen consumption and mitochondrial metabolisms. (D) The motif represents for the H3K9me2 specific unique peaks observed in DMSO samples, which is lost in the 27‐HC treated cells.

**FIGURE 5:**
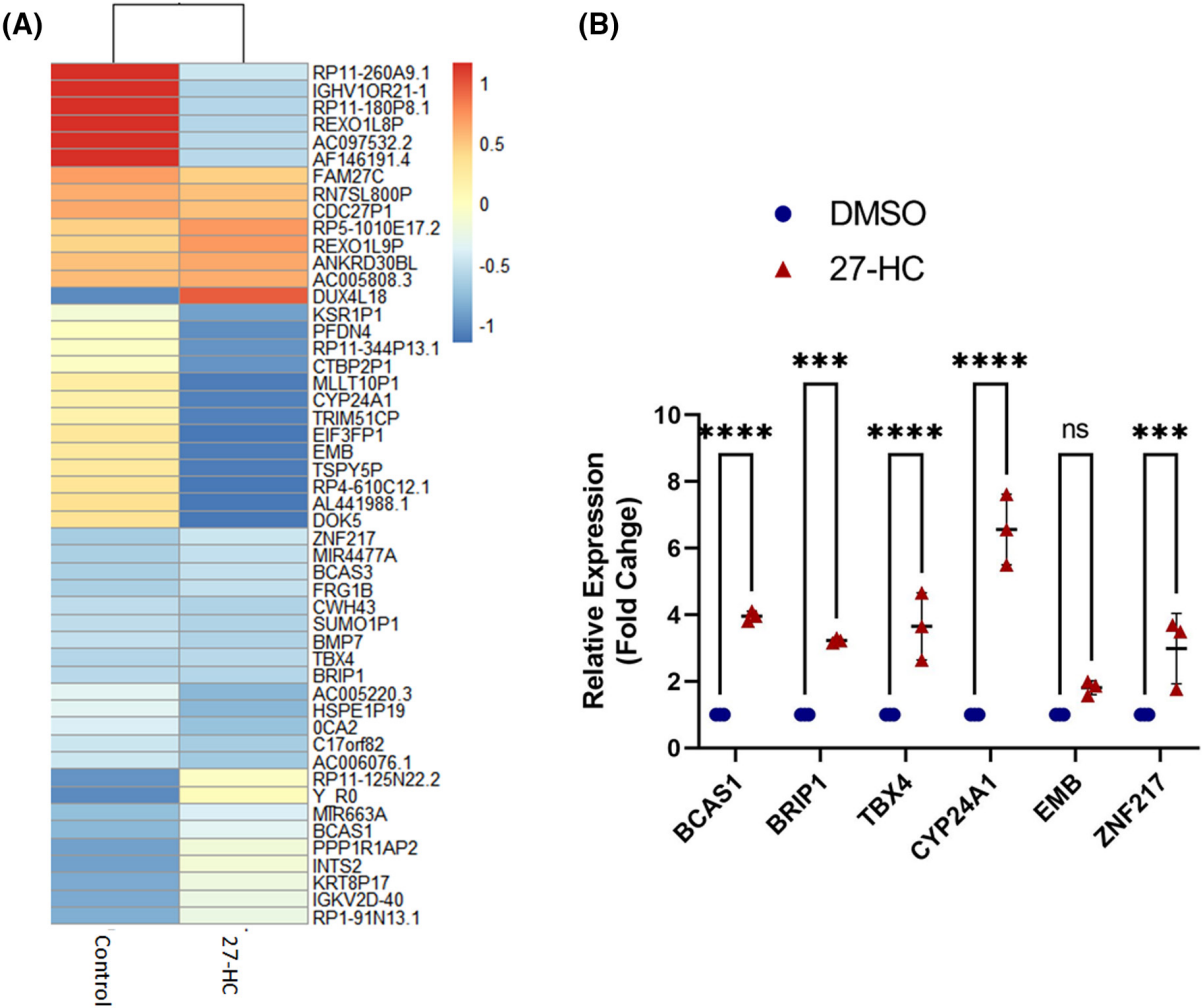
Identification of the differentially regulated G9a target genes in 27‐HC treated cells by global H3K9me2 ChIP sequencing. (A) Represents heatmap of fold enrichment values for the top 50 genes across Control and Test samples. Heatmap R package was used for heatmap generation for selected genes. (B) The graph represents the qRT‐PCR analysis for *BCAS1*, *BRIP1*, *TBX4*, *CYP24A1* and EMB, genes which showed H3K9 dimethylation mark reduction. An upregulation of expression of these genes was observed in 27‐HC treated cells. The GAPDH was used as normalisation control and the error bar represents the standard deviation of three independent replicates. The *p* values were calculated using two‐way anova test and the differences were considered significant at *p* values <0.05(*), <0.01(**), <0.001(***).

Upon 27‐HC treatment, a decrease in G9a and H3K9me2 were observed in MCF‐7, prompting the analysis of global alternations in H3K9me2 mark through ChIP sequencing. An anti‐H3K9me2 mark antibody was used to map the H3K9me2 mark target genes hypomethylated by 27‐HC treatment in MCF‐7 cells. ChIP sequencing peak calling identified a considerable reduction in the number of peaks in 27‐HC treated cells (Figure 4A). The distribution of unique peaks in DMSO‐treated cells signified that most of the peaks were distributed in the intronic and intergenic regions of the genes (Figure 4B), which indicates that the 27‐HC–mediated reduction of H3K9me2 marks is mostly located in these regions and, to some extent, in the promoters. Gene Ontology and motif analysis indicated that ChIP sequencing peaks were predominantly linked to pathways associated with oxygen and mitochondrial metabolism (Figure 4C). H3K9me2‐specific unique peaks were observed in DMSO treated cells, which were lost upon 27‐HC treatment (Figure 4D). The 27‐HC–mediated changes in H3K9me2 distribution might have altered the subsets of pathways related to mitochondrial metabolism in cancer cells. The set of genes that preferentially lost the H3K9me2 mark upon 27‐HC treatment were viz. breast cancer 1, early onset (*Breast cancer gene 1 (BRCA1*)‐interacting protein 1 (*BRIP1*), breast carcinoma amplified sequence 1 (*BCAS1*), *MIR663A*, zinc‐finger protein 217 (*ZNF217*), T‐box transcription factor (*TBX4*), docking protein 5 (*DOK5*), embigin (*EMB*), breast carcinoma amplified sequence 3 (*BCAS3*), bone morphogenetic protein 7 (*BMP7*), vitamin D 24‐hydroxylase (*CYP24A1*) and *Y_RNA* (Figure 5A). Of these genes, *BRIP1*, *BCAS1*, *MIR663A*, *ZNF217*, *TBX4*, *DOK5* and *EMB* experienced significant loss of H3K9 dimethylation in 27‐HC–treated MCF‐7 cells (Figure 5A). BRIP1 and CYP24A1 are involved in mitochondrial metabolism. Interestingly, all these genes were linked to cancer progression by either involving in, invasion and metastasis, or stemness.^31^, ^32^, ^33^, ^34^, ^35^, ^36^, ^37^, ^38^ As H3K9me2 is generally a gene repressor mark, the expressions of selected ChIP target genes were tested using qRT‐PCR. The expression levels of *BRIP1*, *BCAS1*, *TBX4*, *EMB* and *CYP24A1* were analysed, which showed that the expressions of *BRIP1*, *BCAS1*, *TBX4* and *CYP24A1* were upregulated 2.5, 3.4, 5.8 and 5.9 folds, respectively (Figure 5B). Hence, it is evident that 27‐HC regulates a subset of cancer progression genes via modulation of the euchromatic histone lysine methyltransferase G9a to deregulate the H3K9me2 mark in MCF‐7 cells.

### 3.5 Correlation of the 27‐HC catabolising enzyme CYB7B1 and G9a expression in breast cancer tissues

**FIGURE 6:**
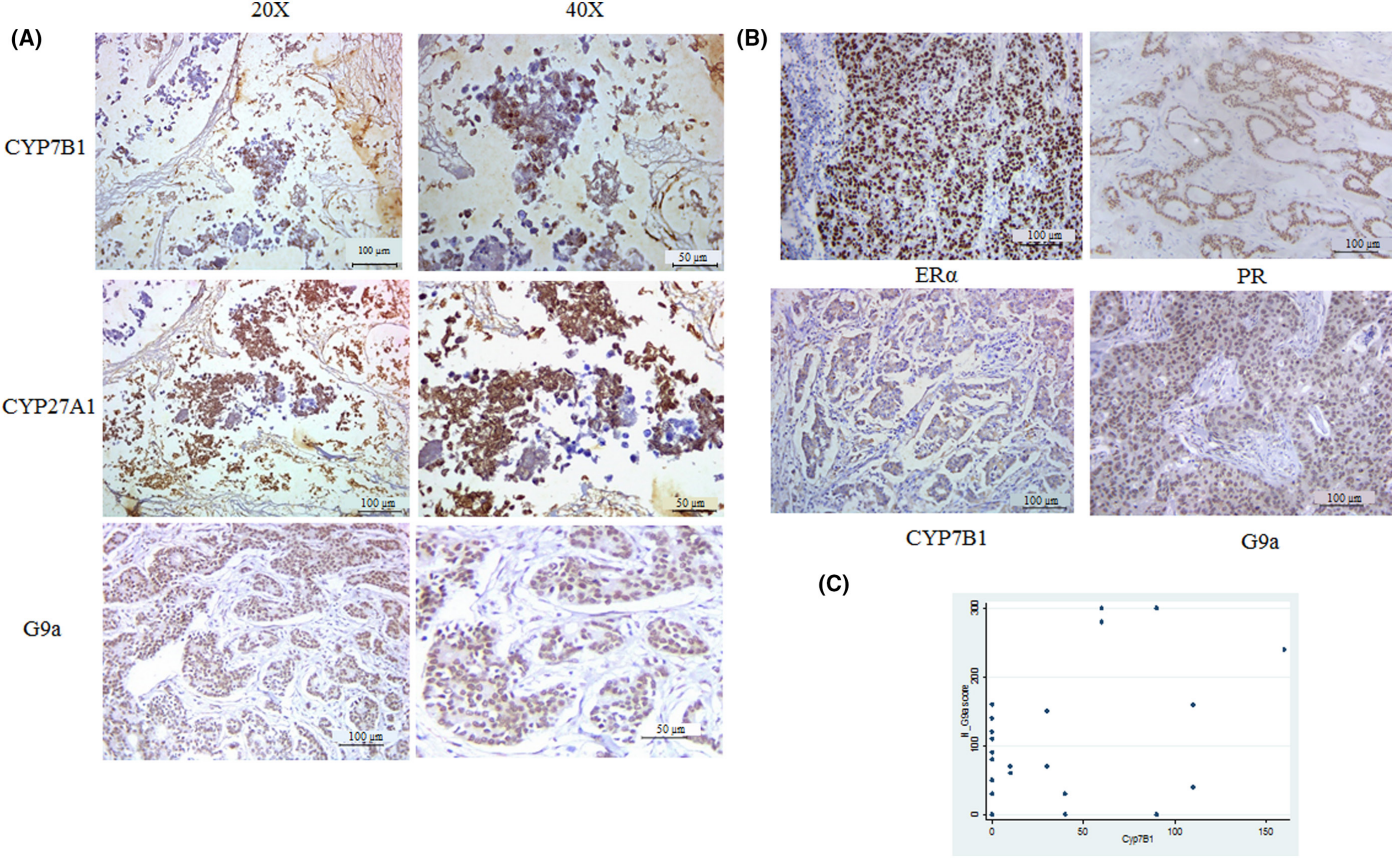
Expression of CYP7B1, CYP27A1 and G9a in ER positive breast cancer patient samples: Tissue microarray was constructed using 87 breast cancer patient samples and immunohistochemical analysis of CYP7B1, CYP27A1 and G9a were performed. (A, B) Representative images of ER positive breast cancer Tumours stained for CYP7B1, CYP27A1, G9A by immunohistochemical analysis at magnification of 20× and 40×. (C) The graph represents the correlation between CYP7B1 and G9a. A moderate positive correlation (*r* = 0.4065, *p* = 0.0189) was observed between H score of G9a and CYP7B1 in pre‐menopausal group.

The expressions of key enzymes involved in 27‐HC metabolism, namely, CYP27A1 and CYP7B1,^9^ in breast tissues and their correlation with G9a were assessed in both premenopausal and postmenopausal women with breast cancer of tumour grade 2 and grade 3. CYP27A1 synthesizes 27‐HC, whereas CYP7B1 catabolizes 27‐HC,^9^ thus reducing its levels. Of the 87 samples from patients with breast cancer, 68 were ER‐positive and 19 were ER‐negative. Of these, 36 belonged to tumour grade 2 and 51 to tumour Grade 3. A correlation analysis was performed between different groups using Pearson correlation. This analysis indicated a correlation among G9a, CYP7B1 in premenopausal category of women (Figure 6A,B) (Tables S2–S4). Secondary antibody controls were used to ensure that the observed IHC signals in the tissues were specific for CYPB1 and G9a proteins (Figure S1). A moderately significant correlation of G9a (nuclear) with CYP7B1 (*r* = 0.42, *p* = 0.05) and a mild negative correlation with CYP27A1 (*r* = −0.32, *p* = 0.15) in the category of premenopausal women were observed irrespective of their hormonal status (Figure 6C). This pattern was also seen in cytoplasmic expression of G9a, which demonstrated a moderate positive correlation with CYP7B1 (*r* = 0.43, *p* = 0.04) in the premenopausal category. The expressions of each of the variables, namely, G9a nuclear, G9a cytoplasmic, G9a total, CYP7B1 and CYP27A1, were also analysed with regard to tumour grade and menopausal status using the median test. The data were represented as the median [interquartile range]. A statistically significant difference was observed in the expressions of these proteins between tumour grade and hormonal status. However, a slightly higher expression of CYP27A1 was observed in premenopausal women compared with the postmenopausal category, although not statistically significant. This finding suggests the higher levels of 27‐HC in premenopausal women than in postmenopausal women. A similar pattern was also observed in the samples of patients with grade 3 tumour.

## 4 DISCUSSION

27‐HC is an endogenous SERM known to augment ER‐dependent breast cancer proliferation and metastasis via the LXR‐dependent pathway.^12^ However, not much is known regarding the modulation of the epigenetic machinery by 27‐HC and its contribution to the aberrant epigenome, which can alter the normal or tumour cells and aid them in attaining higher proliferative capacity, thus resulting in tumorigenesis. In this study, 27‐HC was identified to reduce the expression of the euchromatic histone lysine methyltransferase G9a in ER‐positive MCF‐7 breast cancer cells. G9a/EHMT2 belongs to the SET domain‐containing Su(var)3–9 family of proteins that transfers methyl groups from S‐adenosyl‐l‐methionine to the ε‐amino group of the target lysine residue, resulting in the monomethylation and dimethylation of H3K9 and, to a lesser extent, H3K27.^39^ G9a has been shown to act either as a repressor or as an activator depending upon its interacting partners.^40^ G9a has been observed to be mutated or amplified at a low frequency in various tumours and has been described as an oncogene or a tumour suppressor.^40^, ^41^ G9a repression has been reported to be associated with self‐renewal^42^ and highly tumorigenic tumour‐propagating or cancer stem cells.^43^ Furthermore, unlike their wild‐type counterparts, G9a‐depleted tumours develop after a prolonged latency. Nevertheless, these tumours are more aggressive and have an expanded cancer progenitor pool and pronounced genomic instability.^41^ In this study, reduced levels of G9a and H3K9 dimethylation were observed, which might induce epigenetic reprogramming to stimulate the proliferative and invasive capacity of cancer cells.

ChIP assay demonstrated the reduced occupancy of ERα at the G9a promoter. Although oestrogen drives breast cancer proliferation, the molecular mechanisms of ligand‐bound ERα and its effects on gene regulation and chromatin organisation remain poorly understood. There is limited understanding of the hierarchical events that occur at the genomic and epigenomic levels hours after exposure to oestrogen.^16^ ERα‐mediated transcriptional repression includes the physiological squelching of coactivator proteins and the involvement of components of the basal transcriptional machinery.^44^ Moreover, polycomb complexes are recruited to enhance the inhibiting interaction with p300/CBP, thus preventing transcriptional progression. ERα has also been reported to repress genes by targeting DNMT3B.^45^ We have previously shown that 27‐HC can induce DNA hypermethylation on the promoters of a subset of genes via the de novo DNA methyltransferase DNMT3B in MCF7 cells.^17^ ChIP results allude that 27‐HC mediated the reduced ERα occupancy at the *G9a* promoter, which resulted in the transcriptional silencing of *G9a*. Furthermore, G9a has been shown to act as a coactivator of ERα.^46^ G9a reduction by 27‐HC was found to decrease p21 at the transcriptional and translational levels, which agrees with the earlier findings that G9a activates p21 expression.^25^ p21 is known to act as a double‐edged sword in cancer cells depending on cell type, cellular localisation, p53 status and the type and level of genotoxic stress.^28^, ^29^, ^47^

27‐HC reduced ERα, which is consistent with a previous report.^30^ Moreover, MG132 pretreatment prevented 27‐HC–mediated ERα degradation. Additionally, the ERα antagonists fulvestrant and the SERM 4‐hydroxytamoxifen, in combination with 27‐HC, induced upregulation of *G9a* gene expression. Furthermore, the fact that ERα silencing caused the reduction of G9a confirmed that 27‐HC–mediated G9a repression is mediated by ERα. ChIP sequencing of the H3K9me2 mark identified reduced signal in a subset of genes in 27‐HC–treated cells (Figure 5A). These genes include *ZNF217*, *CYP24A1*, *BRIP1*, *BCAS1*, *EMB* and *TBX4*. Of these, *BRIP1, ZNF217, BCAS1* and *TBX4* were significantly upregulated in MCF‐7 cells upon 27‐HC treatment. These genes were linked to cancer progression.^32^, ^33^, ^34^, ^35^, ^36^, ^37^, ^38^, ^48^, ^49^

A moderate correlation was observed between G9a and CYP7B1 in patients belonging to the premenopausal age group. Statistically significant expression changes were not noted in CYP7B1 and CYP27A1 between menopausal status and tumour grade. A slightly higher expression of CYP27A1 was observed in premenopausal women than in postmenopausal women. Additionally, when CYP27A1, the 27‐HC synthesising enzyme was higher, 27‐HC was likely to be higher, G9a showed a mild negative correlation (−0.3229), although not statistically significant. This observation agrees with our in vitro study in which 27‐HC downregulated G9a expression. Also, in postmenopausal women, the expressions of CYP7B1 and CY27A1 tend to be lower than those in postmenopausal women. The activity of these enzymes and their expression ratios may decide the level of 27‐HC. Elevated tumoral mRNA expression of *CYP7B1* has also been reported to be associated with increased recurrence‐free survival.^8^ The Cancer Genome Atlas data analysis showed that CYP27A1 levels are more or less similar in breast tumours and normal breast tissues, whereas the CYP7B1 expression is significantly lower in breast tumours.^50^ CYP7B1 has also been shown to be hypermethylated in breast tissues, and the recruitment of monocytes to breast tissues is likely to enhance the accumulation of 27‐HC.^51^ In this study, increased CYP7B1 expression was associated with increased G9a. 27‐HC levels in the serum were not assessed in this study as these levels are not correlated with the levels in breast tumours, unlike normal breast tissues.^12^ Assuming that the levels of 27‐HC are lower in the breast tissues as CYP7B1 is higher, the higher G9a correlates well with our observed cellular results.

In this study, a statistically significant difference was not obtained between nuclear and cytoplasmic G9a. However, the median *H*‐score of G9a in the cytoplasm tends to be higher than that in the nucleus. Although G9a was previously thought to be localized primarily in the nucleus, accumulating evidence indicates that the G9a protein is localized both in the nucleus and in the cytoplasm. The localisation of endogenous G9a in the cytoplasmic compartment requires the exclusion of exon 10 (E10) from the *G9a* mRNA.^52^ G9a has been reported to shuttle between the nucleus and the cytoplasm).^53^ Interestingly, G9a cytoplasmic intensity, but not nuclear intensity, in oral squamous carcinoma cells was associated with histological grade of differentiation (tumour grade).^54^ Further studies and analysis using larger patient samples are required to determine whether G9a plays similar roles in breast cancer. In addition, studies are essential to understand the molecular basis of 27‐HC–mediated reduction in the expression of G9a and breast cancer development. G9a depletion has been reported to differentially affect the expressions of ERα target genes.^55^ This effect may result in a chain of events, including the stimulation of genes responsible for cancer proliferation and invasion and suppression of tumour suppressors. Many of these events may cumulatively lead to breast cancer progression.

## AUTHOR CONTRIBUTIONS

**Ravindran Vini:** Conceptualization (equal); formal analysis (lead); investigation (lead); writing – original draft (lead). **Asha Lekshmi:** Formal analysis (supporting); investigation (supporting). **Swathy Ravindran:** Investigation (supporting). **Jissa Vinoda Thulaseedharan:** Formal analysis (supporting). **Kunjuraman Sujathan:** Formal analysis (supporting). **Arumugam Rajavelu:** Conceptualization (equal); formal analysis (equal); supervision (equal); writing – review and editing (equal). **Sreeharshan Sreeja:** Conceptualization (equal); formal analysis (equal); funding acquisition (equal); supervision (equal); writing – review and editing (equal).

## FUNDING INFORMATION

This work was supported by RGCB intramural support and ICMR extramural funding (RFC No. NCD/Ad‐hoc/51/2021–22) and Department of Biotechnology (DBT) (No. BT/01/CEIB/01/CB/2016). VR is supported by ICMR Senior Research Fellowship, Govt of India.

## CONFLICT OF INTEREST STATEMENT

The authors declare that they have no conflict of interest with the contents of this article.

## Supporting information


Data S1:

## Data Availability

All the data generated in this study are included in the manuscript. The NGS files related to H3K9me2 ChIP sequencing are generated in this study are available for download from NCBI GEO accession number: GSE232895.
