# Supplementary material for: 27‐Hydroxycholesterol represses G9a expression via oestrogen receptor alpha in breast cancer
Source: J Cell Mol Med. 2023 Aug 23;27(18):2744–55. doi: 10.1111/jcmm.17882 (PMC10494299; doi:10.1111/jcmm.17882)
Supplement: Supplementary file 1 — Data S1: [file JCMM-27-2744-s001.docx]

**Supplementary information for**

**27‐Hydroxycholesterol represses G9a expression via oestrogen receptor alpha in breast cancer**

Ravindran Vini^1,2#^, Asha Lekshmi^3#^, Swathy Ravindran^1^, Jissa Vinoda Thulaseedharan^4^, Kunjuraman Sujathan^3,5^*, Arumugam Rajavelu^1,6^*, Sreeharshan Sreeja^1^*

1. Cancer Research Program, Rajiv Gandhi Centre for Biotechnology (RGCB), Thycaud PO, Thiruvananthapuram, Kerala, 695014, India.
2. Research Centre, University of Kerala, Thiruvananthapuram, Kerala.
3. Laboratory of Cytogenetics and Molecular Diagnostics, Division of Cancer Research, Regional Cancer Centre, Thiruvananthapuram, Kerala
4. Achutha Menon Centre for Health Science Studies (AMCHSS), Sree Chitra Tirunal Institute for Medical Sciences and Technology, Thiruvananthapuram- Kerala.
5. Health Software Technology Group, Centre for Development of Advanced Computing (CDAC), Thiruvananthapuram, Kerala.
6. Department of Biotechnology, Bhupat & Jyoti Mehta School of Biosciences, Indian Institute of Technology Madras, Chennai, Tamil Nadu, 600 036, India.

# First author

*Corresponding authors

Correspondence to be addressed: [ksujathan@gmail.com](mailto:ksujathan@gmail.com); [arumugam.rajavelu@iitm.ac.in](mailto:arumugam.rajavelu@iitm.ac.in); [ssreeja@rgcb.res.in](mailto:ssreeja@rgcb.res.in)

**Supplementary figure 1:**

**
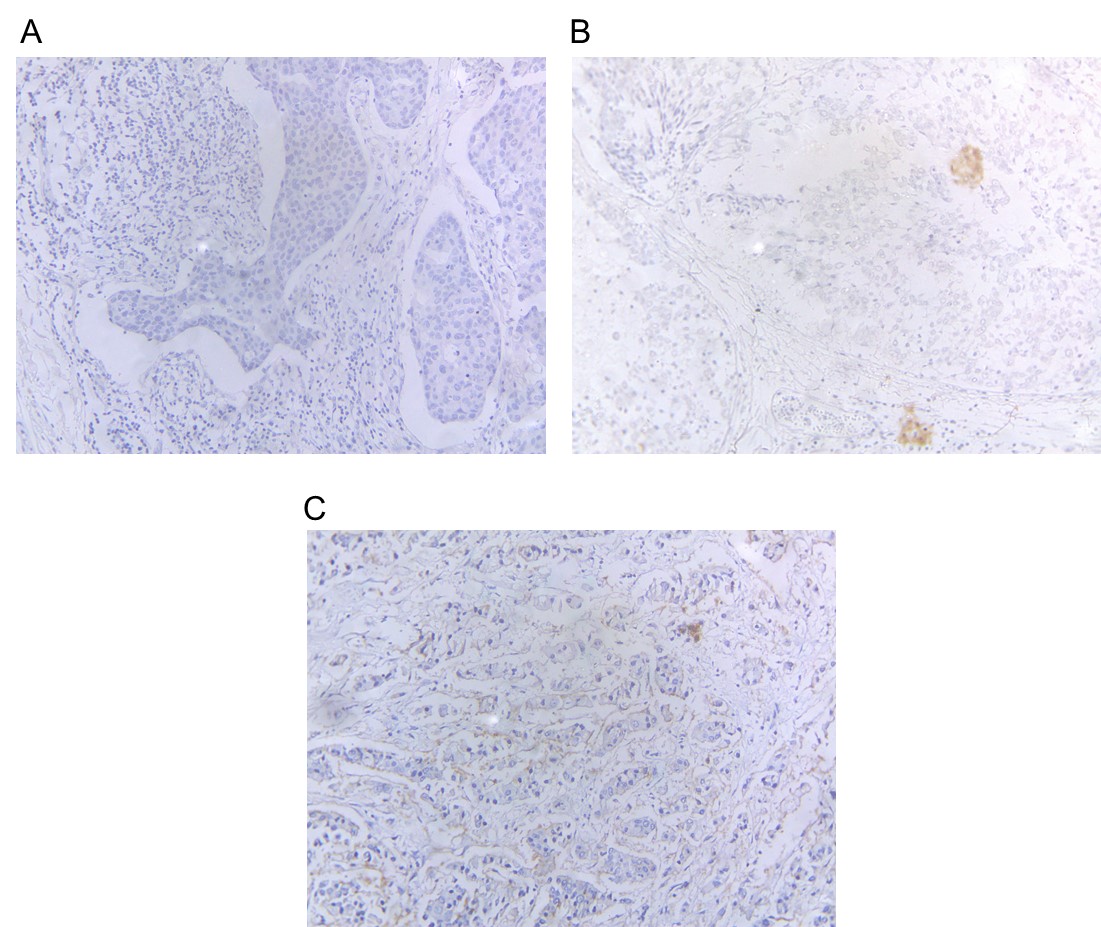
**

**Supplementary figure 1:** The representative images for the secondary antibody staining for (A) CYP7B1, (B) CYP27A1, and (C) G9a antibodies indicate that the observed signal for the primary antibodies (Figure 6) are specific to each protein.

**Supplementary figure 2:**

**
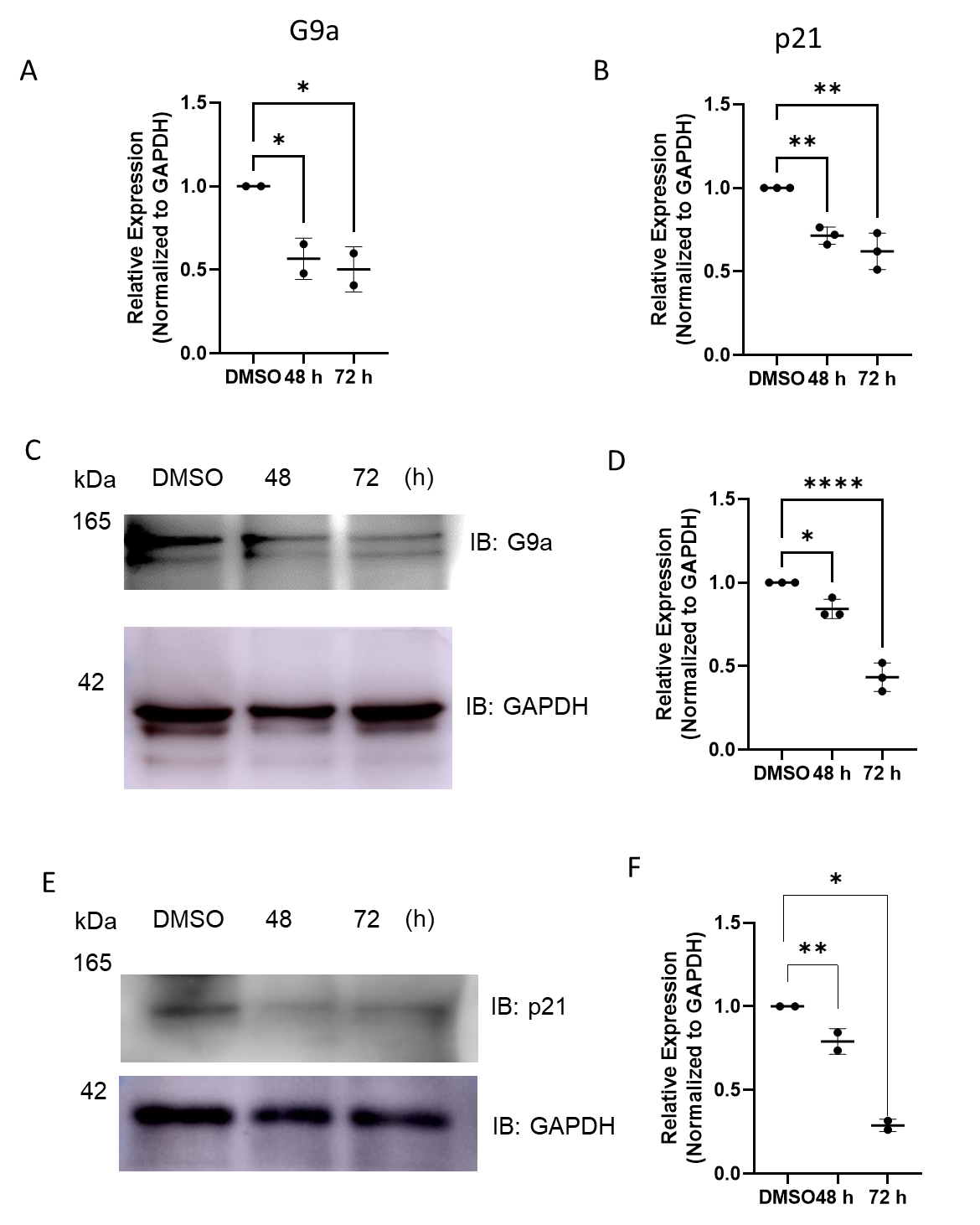
**

**Supplementary figure 2: Downregulation of G9a expression and its target protein p21 upon 27-HC treatment in T47D cells. (A)** The graph represents the qRT-PCR analysis for expression of *EHMT2*/*G9a* which showed downregulation upon 27-HC treatment. (**B**) *p21*, a non-histone target of G9a was checked for its expression by qRT-PCR and seen to decrease. **(C)** Protein expression analysis by western blot showed a reduction of G9a upon 27-HC treatment (1 µM). **(D)** The graph represents the intensity of G9a normalised with intensity of corresponding GAPDH. (**E**) p21 protein was seen to decrease consistent with transcriptional level. (**F**) The graph represents the intensity of p21 normalised with intensity of corresponding GAPDH levels. The western blot and qRT-PCR were performed in biological triplicates. The intensity of bands in western blot analysis were measured using ImageJ. The p values were calculated using one-way ANOVA and the differences were considered significant at p values p <0.05(*) <0.01(**) <0.001(***).

**Supplementary figure 3:**

**
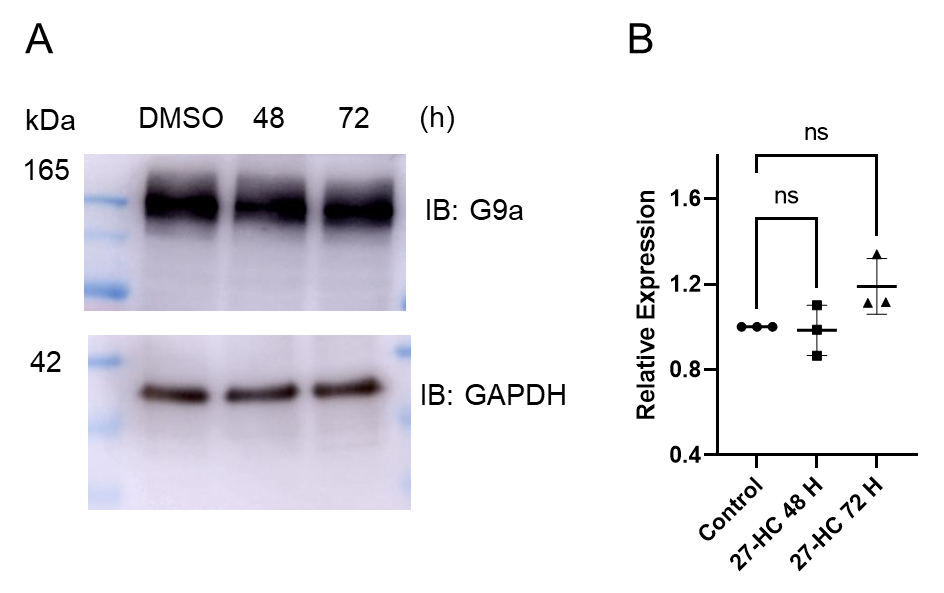
**

**Supplementary figure 3: G9a expression upon 27-HC treatment in ER negative cell line MDA MB- 231. (A)** Protein expression analysis by western blot did not any show significant change in the G9a expression upon 27-HC treatment (1 µM). **(B)** The graph represents the intensity of G9a normalised with intensity of corresponding GAPDH. The intensity of bands in western blot analysis were measured using ImageJ. The p values were calculated using one-way ANOVA and the differences were considered significant at p values p ≥0.05 was treated as non-significant (n s).

**Supplementary table 1:**

**List of primers**

| **S. No** | **Gene** | **Primer sequence (5' to 3')**  **mRNA** |
| --- | --- | --- |
|  | *HAT1* | F.P: TATTGCTGGTAGCCTGTCAAC  R.P: CCTCAACATCATCTGCCTCTAC |
|  | *MYST2* | F.P: TGAGGAGCCTGCTTACTCTA  R.P: GTCTGCCGAAGAGGGTATTT |
|  | *HDAC1* | F.P: GGGATTGATGACGAGTCCTATG  R.P: GAGTCTGAGCCACACTGTAAG |
|  | *HDAC6* | F.P: GGAGGGTCCTTATCGTAGATTG  R.P: GTAGCGGTGGATGGAGAAATAG |
|  | *HDAC7* | F.P: CCTGGTGTCTGCTGGATTT  R.P: TCAGTTGCTGCGTCATGTAT |
|  | *DNMT1* | F.P: TGGCTTTGATGGAGGTGAAA  R.P: CTCCTGCATCAGCCCAAATA |
|  | *DNMT3a* | F.P: CTGAGGTAGCGACACAAAGTTA  R.P: CTCTTCTGGGTGCTGATACTTC |
|  | *DNMT3b* | F.P: GGAGCCACGACGTAACAAATA  R.P: GTAAACTCTAGGCATCCGTCATC |
|  | *SET9* | F.P: TTCACTCCAAACTGCATCTACGA  R.P: GGGTGCGGATGCATTTG |
|  | *SMYD3* | F.P: CCCAACTGTTCGATTGTGTTCA  R.P: TCCTCTCCCACCTCGATGTC |
|  | *JMJD1C* | F.P: ACGGAGATGTGGAGACCTAATA  R.P: CTAGAAGATGGACGCACAATGA |
|  | *LSD1* | F.P: CTAATGCCACACCTCTCTCAAC  R.P: CACACGAGTAGCCATTCCTTAC |
|  | *SUV39H* | F.P: CTGCCCATCTACGAGTGCAA  R.P: TACCCTTCTGTACCACACGATTTG |
|  | *SETDB1* | F.P: GACTCTCTGAGACAACTTCCAAGGA  R.P: CAGGGATTGAGGGAGGAACA |
|  | *G9A* | F.P: CTCCGCTGATTTTCGAGTGTAA  R.P: CTCTGTACGACCCGGTTCTTG |
|  | *EZH2* | F.P: CAAGCAGTGCCCGTGCTA  R.P: AGCGGCTCCACAAGTAAGACA |
|  | *DOT1L* | F.P: CATCCGATGGGTCTGTGA  R.P: TGGTGTCATAGTCAATTAAAACGTAATTC |
|  | *MLL1* | F.P: GAGGACCCCGGATTAAACAT  R.P: GGAGCAAGAGGTTCAGCATC |
|  | *MLL2* | F.P: AGGAGCTGCAGAAGAAGCAG  R.P: CAGCCAAACTGGGAGAAGAG |
|  | *SETD2* | F.P: ACGTCATGGGCTGTCTTTGT  R.P: TTGGGAGTGTCTGTGTCAGC |
|  | *ZNF217* | F.P: CAGCGAGGTCGATTCTCCAA  R.P: TTTCTCTTTTGTGCCATGCTGT |
|  | *CYP24A1* | F.P: TGGGTTCCTTTGAGTCGGTG  R.P: CGATAGGCCTTCCACGGTTT |
|  | *BRIP1* | F.P: TTGCTTTTGTTCCAGAACGGT  R.P: CATCGTTCCCTAAATGTAAATGAGT |
|  | *BCAS1* | F.P: TGGGTAGAATACTTGGGGTGC  R.P: GGTTCTCTTCCTGATGTGGGG |
|  | *EMB-1* | F.P: AATCTCTGTAGGCTCACCTTTG  R.P: GGGTTAGTCAAGCAGGTAGAAG |
|  | *TBX4* | F.P: GGAAAGGGTCATGGGCTAAA  R.P: GGGAAGGGCTCTGTACTTTATT |
|  | *DOK 5* | F.P: GTGTGTAGGAACACGGATCAA  R.P: GGACGTCCCAAAGACAGATATAC |
|  | *GAPDH* | F.P: TGCACCACCAACTGCTTAGC  R.P: GGCATGGACTGTGGTCATGAG |
|  | *EHMT2 PS1* | F.P: CAGTATTTTTTAAGAAAAAAC  R.P: CCATGTTTAGGGCTGGGCTCCT |
|  | *EHMT2* PS2 | F.P: GTCTTCAGGAAAGCTAAAAAA  R.P: TTAGGTATTACGTTTTGGTCG |
|  | *EHMT2* PS3 | F.P: GGTACCCTAAATACAGCTATCG  R.P: CACTAGGGATCCAGGTAAGCCC |

**The primers from S. No 28-30**: *EHMT2/*G9a promoter primers for Chip -qPCR. PS1, PS2, PS3 are primers specific to different regions of promoter of G9 gene. The ChIP-qPCR result representation in the manuscript is using *EHMT2* PS2. . There was no statistically significant difference in enrichment of ERα on other regions of *EHMT2* promoter in 27-HC treated cells when compared to DMSO treatment.

**Supplementary table 2:** Shows range of parameters analysed by IHC in tissue samples

|  | **Age** | **G9a Nuclear** | **G9a Cyto** | **G9a** | **CYP7B1** | **CYP27A1** |
| --- | --- | --- | --- | --- | --- | --- |
| **Minimum** | 28 | 0 | 0 | 0 | 0 | 0 |
| **25th Percentile** | 45 | 0 | 0 | 0 | 0 | 0 |
| **50th Percentile** | 55 | 20 | 60 | 70 | 0 | 15 |
| **75th Percentile** | 62 | 80 | 80 | 160 | 15 | 55 |
| **Maximum** | 84 | 200 | 160 | 300 | 160 | 160 |

**Supplementary table 3:** Shows the Median test analysis of each protein with respect to their tumour grade status which is represented as Median [Interquartile range]

|  | **Tumour Grade** | |  |
| --- | --- | --- | --- |
| **Grade** | 2 | **3** | **P-Value** |
| **G9a Nuclear** | 35[0-75] | 0[0-80] | 0.503 |
| **G9a Cytoplasmic** | 70[0-80] | 60[0-80] | 0.356 |
| **G9a Total** | 95[15-155] | 70[0-160] | 0.239 |
| **CYP7B1** | 0[0-0] | 0[0-40] | 0.256 |
| **CYP27A1** | 0[0-50] | 20[0-60] | 0.652 |

**Supplementary table 4:** Shows the Median test analysis of each protein with respect to their hormonal status which is represented as Median [Interquartile range]

|  | **Menopausal Status** | |  |
| --- | --- | --- | --- |
|  | **Premenoapuse** | **Postmenopause** | **P-Value** |
| **G9a Nuclear** | 10[0-80] | 25[0-80] | 0.619 |
| **G9a Cytoplasmic** | 60[30-60] | 65[0-80] | 0.835 |
| **G9a Total** | 70[30-160] | 80[0-160] | 0.936 |
| **CYP7B1** | 0[0-40] | 0[0-0] | 0.104 |
| **CYP27A1** | 25[0-50] | 0[0-55] | 0.241 |
